# Molecular Detection and Genetic Characterization of *Toxoplasma gondii* in Farmed Minks (*Neovison vison*) in Northern China by PCR-RFLP

**DOI:** 10.1371/journal.pone.0165308

**Published:** 2016-11-02

**Authors:** Wen-Bin Zheng, Xiao-Xuan Zhang, Jian-Gang Ma, Fa-Cai Li, Quan Zhao, Si-Yang Huang, Xing-Quan Zhu

**Affiliations:** 1 State Key Laboratory of Veterinary Etiological Biology, Key Laboratory of Veterinary Parasitology of Gansu Province, Lanzhou Veterinary Research Institute, Chinese Academy of Agricultural Sciences, Lanzhou, Gansu Province, PR China; 2 College of Animal Science and Technology, Jilin Agricultural University, Changchun, Jilin Province, PR China; 3 Jiangsu Co-innovation Center for the Prevention and Control of Important Animal Infectious Diseases and Zoonoses, Yangzhou University College of Veterinary Medicine, Yangzhou, Jiangsu Province, PR China; Institut national de la santé et de la recherche médicale - Institut Cochin, FRANCE

## Abstract

*Toxoplasma gondii* is a worldwide prevalent parasite, affecting a wide range of mammals and human beings. Little information is available about the distribution of genetic diversity of *T*. *gondii* infection in minks (*Neovison vison*). This study was conducted to estimate the prevalence and genetic characterization of *T*. *gondii* isolates from minks in China. A total of 418 minks brain tissue samples were collected from Jilin and Hebei provinces, northern China. Genomic DNA were extracted and assayed for *T*. *gondii* infection by semi-nested PCR of B1 gene. The positive DNA samples were typed at 10 genetic markers (SAG1, SAG2 (5'+3' SAG2, alter.SAG2), SAG3, BTUB, GRA6, c22-8, c29-2, L358, PK1, and Apico) using polymerase chain reaction-restriction fragment length polymorphism (PCR-RFLP) technology. 36 (8.6%) of 418 DNA samples were overall positive for *T*. *gondii*. Among them, 5 samples were genotyped at all loci, and 1 sample was genotyped for 9 loci. In total, five samples belong to ToxoDB PCR-RFLP genotype#9, one belong to ToxoDB genotye#3. To our knowledge, this is the first report of genetic characterization of *T*. *gondii* in minks in China. Meanwhile, these results revealed a distribution of *T*. *gondii* infection in minks in China. These data provided base-line information for controlling *T*. *gondii* infection in minks.

## Introduction

*Toxoplasma gondii* is an opportunistic pathogen, which can infect virtually all warm-blooded animals and humans [1]. An estimated one-third of the world populations and approximately 8% of Chinese people were infected with *T*. *gondii* [1,2]. Humans and animals get infection mainly through ingestion of tissue cysts in undercooked meat or oocysts in food or water [3]. *T*. *gondii* infection can present with various symptoms in humans and animals [4–7]. Generally, *T*. *gondii* infection rarely causes clinical symptoms in healthy individuals, however, it can cause severe disease, even fatal to AIDS patients or those individuals with cancer undergoing immuno-suppressive therapy [2].Moreover, primo infection with *T*. *gondii* during pregnancy may cause severe damages to the fetus by transplacental transmission [8,9].

Recently, a number of studies have been focusing on *T*. *gondii* genotypes in a variety of hosts, including humans [3,10,11], the vast majority of *T*. *gondii* strains in North America and Europe are mainly classified into four clonal lineages (Types I, II, III and 12), whereas, *T*. *gondii* isolates in Africa and South America are more genetically diverse. Thus, it is necessary to determine genetic characterization of *T*. *gondii* isolates from different sources in China. China is a biodiversity-rich country. Limited information on *T*. *gondii* genotypes has been reported in patients, pets, food animals, poultry, birds and several species of wild animals [3,12–16]. The genotype ToxoDB#9 is predominant in different parts of China. Although the amount of data on *T*. *gondii* genotypes is growing, the information regarding the distribution of *T*. *gondii* genotypes is limited in China, and it is yet to know *T*. *gondii* genotypes in minks, one of the most important fur-bearing animals.

Minks is not only an important economic animal, but also become an important source of *T*. *gondii* infection for humans and other animals. Infected minks could be preyed on by cats, so they are also an important transmission resource. This study, therefore, was conducted to determine the genetic characterization of *T*. *gondii* isolates in minks in China.

## Methods

### Ethics statement

This study was approved before its commencement by the Ethics Committee of the Lanzhou Veterinary Research Institute, Chinese Academy of Agricultural Sciences.

### Sample collection

**Fig 1:**
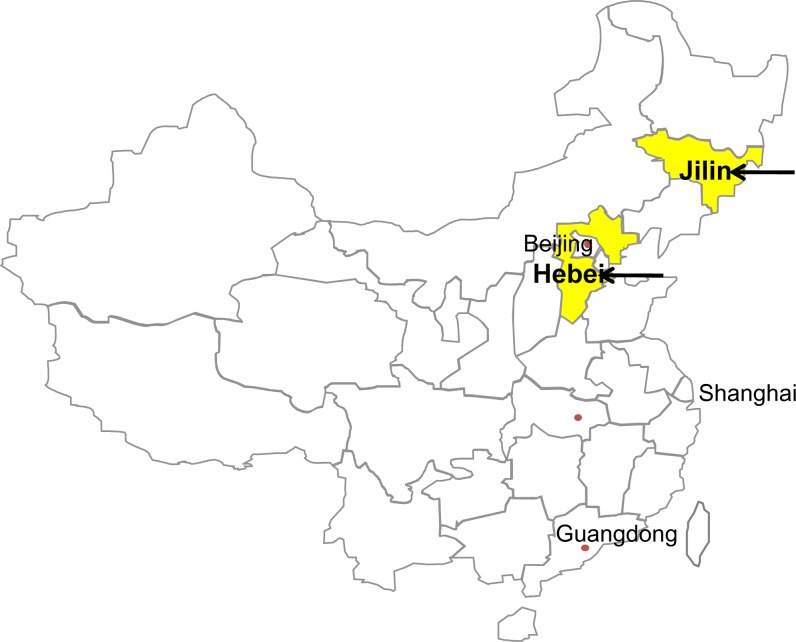
A map of the People’s Republic of China. Arrow indicated areas are the sampling locations for the present study, Jilin and Hebei provinces.

Brain tissue samples were collected from 418 slaughtered minks in Jilin and Hebei province during the slaughter seasons in March 2015 (Fig 1). Of these, 194 (24 HedlundMink (*Mustela vison*) and 170 Jet Black Mink (*Mustela vison*)) were collected from slaughterhouse in Changchun city, the capital of Jilin province, and 224 (98 Jet Black Mink and 126 PalominoMink) were collected from slaughterhouse in Shijiazhuang city, the capital of Hebei province (S1 Table). The minks were killed for furs with knives by the farmers. The collected brain tissues were grinded by liquid nitrogen and stored at -20℃until further analysis.

### Extraction of genomic DNA and genetic characterization

Genomic DNA was extracted directly from each sample by using a commercial DNA extraction kit (TianGen™, Beijing, China). Briefly, 30 mg of the brain tissues samples were treated with proteinase K at 56°C for 12 h in a thermostatic water bath, and subsequent column purification according to manufacturer’s recommendations, and DNA samples were eluted into 60 μl elution buffer. *T*. *gondii* B1 gene was amplified by a semi-nested PCR to detect possible infection in accordance with the previous study [17]. *T*. *gondii*-positive DNA samples were used for genetic characterization. Genotyping was performed using 10 genetic markers (SAG1, SAG2 (5'+3' SAG2, alter.SAG2), SAG3, BTUB, GRA6, c22-8, c29-2, L358, PK1, and Apico) for multiplex multilocus nested PCR-RFLP (Mn-PCR-RFLP) as described previously [3,18–20]. Nine reference *T*. *gondii* strains (GT1, PTG, CTG, MAS, TgCgCa1, TgCatBr5, TgWtdSc40, TgCatBr64 and TgRsCr1) (the DNA samples were provided by Dr. Chunlei Su) were included as controls. Briefly, a total of 25 μl reaction system containing: 1× PCR buffer, 0.2 mM of each primer (S2 Table), 200 μM dNTPs, 2 mM MgCl2, 0.2 U of HotStart Taq DNA polymerase (TAKARA, Japan). The cycling conditions were 95°C for 5 min to activate the DNA polymerase, then 30 cycles of 95°C for 30 s, 55°C for 60 s and 72°C for 90 s. Then 1 μl of the first PCR amplicon served as template DNA for nested PCR with internal primers for each marker, respectively. A similar approach was used for nested PCR. With the exception of Apico (55°C for 60 s) marker, all the markers were annealed at 60°C for 60s. The second PCR products were digested with different restriction enzymes at suitable temperatures for 1.5h. The restriction fragments were observed under UV light after electrophoresis in 2.5%-3% agarose gel containing GoldView^TM^ (Solarbio, China). The results were recorded, compared and matched to those identified RFLP genotypes listed in ToxoDB genotyping database (www.toxodb.org).

### Statistical analysis

Differences in the prevalence of *T*. *gondii*-infected minks among different regions and breeds were analyzed by a Chi-square test using SAS version 9.1 (SAS Institute Inc., USA). If *p* < 0.05, the results were considered statistically significant.

## Results and Discussion

In the present study, 36 (8.6%) of 418 DNA samples were overall positive for *T*. *gondii*, examined by semi-nested PCR, with 8.8% (n = 17) in Jilin province and 8.5% (n = 19) in Hebei province. The prevalence was lower comparing to other studies, such as the 13.9% in farmed minks in Poland by latex agglutination test (LAT) [21], 29.2% in feral minks in UK by PCR [22], 66% in feral minks in USA by the Sabin-Feldman dye test [23], 70% in minks in Chile by LAT [24] and 77% in minks in USA by MAT [25]. The difference may due to the different living environment, regions, the density of felids, the diet of minks and different sensitivity and specificity of detection methods, and the DNA prevalence only reflects prevalence in 2 farms of the two provinces. Although minks from Jilin province (8.8%, OR = 1.04, 95% CI = 0.52–2.06) had a 1.04 fold increase higher risk of being positive compared to that of minks from Hebei province (8.5%), the different was not statistically significant (*P*>0.05). Moreover, PalominoMink (11.9%) were more than three times (OR = 3.11, 95% CI = 0.36–24.72) at risk of acquiring *T*. *gondii* infection compared to HedlundMink (4.2%), indicating that difference of *T*. *gondii* infection could be existence in different breeds of mink, but the difference was also not statistically significant in different breeds (*P*>0.05) (Table 1).

**Table 1:** Analysis of the variables associated with *Toxoplasma gondii* seroprevalence in minks in China.

Variable	Category	No. examined	No. positive	Prevalence % (95% CI)	Variable	Category
Region	Jilin	194	17	8.76 (4.78–12.74)	>0.05	1.04 (0.52–2.06)
	Hebei	224	19	8.48 (4.83–12.13)		Reference
Breeds	HedlundMink	24	1	4.17 (0.00–12.16)	>0.05	Reference
	Jet Black Mink	268	20	7.46 (4.32–10.61)		1.86 (0.24–14.46)
	PalominoMink	126	15	11.91 (6.25–17.56)		3.11 (0.36–24.72)
Total		418	36	8.61 (5.92–11.30)		

OR: odd ratios with 95% confidence intervals.

Due to low DNA concentration, five samples were completely typed at all genetic loci and one sample was typed at 9 markers, showing two genotypes (ToxoDB#3 and ToxoDB#9) (Table 2). A total of five (83.3%, 1 from Jilin province and 4 from Hebei province) samples were identified as ToxoDB#9 which was the predominant genotypes in animals in China according to previous studies [26–31], this result indicated that predominant genotypes of *T*. *gondii* was also prevalent in minks in China. Animals originated from these regions, probably were infected by the predominant strains of *T*. *gondii* in the environment. Moreover, ToxoDB#9 was also be found in free-living *Microtus fortis* [32], in bat [15], in wild waterfowl [14] and in sika deer [27] in Jilin province which suggested that further research should be carried out to investigate the transmission model and route in this district. Four isolates of genotype ToxoDB#9 was also found in Hebei province, this was the first report that *T*. *gondii* isolates was found in this district. Remarkably, one ToxoDB#3 (the Type II variant), so-called QHO isolate, was also found in Heibei province in this study. Genotype ToxoDB#3 was firstly isolated from sheep in Qinghai province [26,33]. ToxoDB#3 was also be found in cats in Yunnan province [34], pet birds and house sparrows in Gansu province [12,35], pigs in Guangdong province[36] and in wild birds in Xinjiang Uygur Autonomous Region [35], indicating that ToxoDB#3 was also prevalent in China. More importantly, the information of *T*. *gondii* isolates in mink was limited worldwide, to our knowledge, only one document recorded that Type II of *T*. *gondii* isolates was found in feral mink in the UK [22] using 5 genetic markers (5' SAG2, 3' SAG2, SAG3, GRA6, BTUB). In China, until the present study, there was no information concerning genetic characterization of *T*. *gondii* isolates in mink. More seriously, the bodies of *T*. *gondii*-infected minks are available food for stray cats, and it is an important source for *T*. *gondii* infection in humans and other animals. The information was fed back to the farmers, which will help them to prevent and control toxoplasmosis. So, it is necessary to pay attention to the *T*. *gondii* infection in minks in China, which is not only for economic contribution, but also for public health.

**Table 2:** Summary of genotyping of *Toxoplasma gondii* in farmed minks in Jilin and Hebei provinces, northern China.

Isolate ID	Host	Tissue	Location	SAG1	5'+3’ SAG2	Alternative SAG2	SAG3	BTUB	GRA6	c22-8	c29-2	L358	PK1	Apico	Genotype
GT1	Goat		United States	I	I	I	I	I	I	I	I	I	I	I	Reference, Type I, ToxoDB #10
PTG	Sheep		United States	II/III	II	II	II	II	II	II	II	II	II	II	Reference, Type II, ToxoDB #1
CTG	Cat		United States	II/III	III	III	III	III	III	III	III	III	III	III	Reference, Type III, ToxoDB #2
MAS	Human		France	u-1*	I	II	III	III	III	u-1*	I	I	III	I	Reference, ToxoDB #17
TgCgCa1	Cougar		Canada	I	II	II	III	II	II	II	u-1*	I	u-2*	I	Reference, ToxoDB #66
TgCatBr5	Cat		Brazil	I	III	III	III	III	III	I	I	I	u-1*	I	Reference, ToxoDB #19
TgWtdSc40	WTD		USA	u-1	II	II	II	II	II	II	II	I	II	I	Type 12, ToxoDB #5
TgCatBr64	Cat		Brazil	I	I	u-1	III	III	III	u-1	I	III	III	I	Reference, ToxoDB #111
TgRsCr1	Toucan		Costa Rica	u-1	I	II	III	I	III	u-2	I	I	III	I	Reference, ToxoDB #52
112	Mink	brain	Jilin, China	u-1	II	II	III	III	II	II	III	II	II	I	ToxoDB #9
247	Mink	brain	Hebei, China	u-1	II	II	III	III	II	II	III	II	II	I	ToxoDB #9
266	Mink	brain	Hebei, China	u-1	II	II	III	III	II	II	III	II	II	I	ToxoDB #9
271	Mink	brain	Hebei, China	u-1	II	II	III	III	II	II	III	II	II	I	ToxoDB #9
380	Mink	brain	Hebei, China	II/III	II	II	II	II	II	II	II	II	II	I	ToxoDB #3
411	mink	brain	Hebei, China	nd	II	II	III	III	II	II	III	II	II	I	ToxoDB #9

* u-1 and u-2 represent unique RFLP genotypes, respectively.

WTD: White-tailed Deer.

nd: not determined.

## Conclusion

The present study revealed that the prevalence of *T*. *gondii* in farmed minks was 8.6% in northern China. Two genotypes (ToxoDB#9 and ToxoDB#3) were firstly identified in minks in China, and this is also the first report about the genetic characterization of *T*. *gondii* in Hebei province China. The results indicated that minks were susceptible to *T*. *gondii* and genetic diversity of *T*. *gondii* exits in minks, which provide fundamental data for controlling *T*. *gondii* infection in minks.

## Supporting Information

S1 Table Data about the minks analyzed. (DOCX)

S2 Table Nested PCR primers used for nested PCR-RFLP of *Toxoplasma gondii* in minks. (DOCX)
