# Supplementary material for: Molecular Detection and Genetic Characterization of Toxoplasma gondii in Farmed Minks (Neovison vison) in Northern China by PCR-RFLP
Source: PLoS One. 2016 Nov 2;11(11):e0165308. doi: 10.1371/journal.pone.0165308 (PMC5091863; doi:10.1371/journal.pone.0165308)
Supplement: S1 Table — (DOCX) [file pone.0165308.s001.docx]

**S1 Table.** Data about the minks analyzed.

| ID | Region | Breeds | Age | Date | Result |
| --- | --- | --- | --- | --- | --- |
| 1 | Jilin | HedlundMink | adult | 2015.3 | Negative |
| 2 | Jilin | HedlundMink | adult | 2015.3 | Negative |
| 3 | Jilin | HedlundMink | adult | 2015.3 | Positive |
| 4 | Jilin | HedlundMink | adult | 2015.3 | Negative |
| 5 | Jilin | HedlundMink | adult | 2015.3 | Negative |
| 6 | Jilin | HedlundMink | adult | 2015.3 | Negative |
| 7 | Jilin | HedlundMink | adult | 2015.3 | Negative |
| 8 | Jilin | HedlundMink | adult | 2015.3 | Negative |
| 9 | Jilin | HedlundMink | adult | 2015.3 | Negative |
| 10 | Jilin | HedlundMink | adult | 2015.3 | Negative |
| 11 | Jilin | HedlundMink | adult | 2015.3 | Negative |
| 12 | Jilin | HedlundMink | adult | 2015.3 | Negative |
| 13 | Jilin | HedlundMink | adult | 2015.3 | Negative |
| 14 | Jilin | HedlundMink | adult | 2015.3 | Negative |
| 15 | Jilin | HedlundMink | adult | 2015.3 | Negative |
| 16 | Jilin | HedlundMink | adult | 2015.3 | Negative |
| 17 | Jilin | HedlundMink | adult | 2015.3 | Negative |
| 18 | Jilin | HedlundMink | adult | 2015.3 | Negative |
| 19 | Jilin | HedlundMink | adult | 2015.3 | Negative |
| 20 | Jilin | HedlundMink | adult | 2015.3 | Negative |
| 21 | Jilin | HedlundMink | adult | 2015.3 | Negative |
| 22 | Jilin | HedlundMink | adult | 2015.3 | Negative |
| 23 | Jilin | HedlundMink | adult | 2015.3 | Negative |
| 24 | Jilin | HedlundMink | adult | 2015.3 | Negative |
| 25 | Jilin | Jet Black Mink | adult | 2015.3 | Negative |
| 26 | Jilin | Jet Black Mink | adult | 2015.3 | Negative |
| 27 | Jilin | Jet Black Mink | adult | 2015.3 | Negative |
| 28 | Jilin | Jet Black Mink | adult | 2015.3 | Negative |
| 29 | Jilin | Jet Black Mink | adult | 2015.3 | Negative |
| 30 | Jilin | Jet Black Mink | adult | 2015.3 | Negative |
| 31 | Jilin | Jet Black Mink | adult | 2015.3 | Negative |
| 32 | Jilin | Jet Black Mink | adult | 2015.3 | Negative |
| 33 | Jilin | Jet Black Mink | adult | 2015.3 | Negative |
| 34 | Jilin | Jet Black Mink | adult | 2015.3 | Negative |
| 35 | Jilin | Jet Black Mink | adult | 2015.3 | Negative |
| 36 | Jilin | Jet Black Mink | adult | 2015.3 | Negative |
| 37 | Jilin | Jet Black Mink | adult | 2015.3 | Negative |
| 38 | Jilin | Jet Black Mink | adult | 2015.3 | Negative |
| 39 | Jilin | Jet Black Mink | adult | 2015.3 | Negative |
| 40 | Jilin | Jet Black Mink | adult | 2015.3 | Negative |
| 41 | Jilin | Jet Black Mink | adult | 2015.3 | Negative |
| 42 | Jilin | Jet Black Mink | adult | 2015.3 | Negative |
| 43 | Jilin | Jet Black Mink | adult | 2015.3 | Negative |
| 44 | Jilin | Jet Black Mink | adult | 2015.3 | Negative |
| 45 | Jilin | Jet Black Mink | adult | 2015.3 | Negative |
| 46 | Jilin | Jet Black Mink | adult | 2015.3 | Negative |
| 47 | Jilin | Jet Black Mink | adult | 2015.3 | Negative |
| 48 | Jilin | Jet Black Mink | adult | 2015.3 | Negative |
| 49 | Jilin | Jet Black Mink | adult | 2015.3 | Negative |
| 50 | Jilin | Jet Black Mink | adult | 2015.3 | Negative |
| 51 | Jilin | Jet Black Mink | adult | 2015.3 | Negative |
| 52 | Jilin | Jet Black Mink | adult | 2015.3 | Negative |
| 53 | Jilin | Jet Black Mink | adult | 2015.3 | Negative |
| 54 | Jilin | Jet Black Mink | adult | 2015.3 | Negative |
| 55 | Jilin | Jet Black Mink | adult | 2015.3 | Negative |
| 56 | Jilin | Jet Black Mink | adult | 2015.3 | Negative |
| 57 | Jilin | Jet Black Mink | adult | 2015.3 | Positive |
| 58 | Jilin | Jet Black Mink | adult | 2015.3 | Positive |
| 59 | Jilin | Jet Black Mink | adult | 2015.3 | Negative |
| 60 | Jilin | Jet Black Mink | adult | 2015.3 | Negative |
| 61 | Jilin | Jet Black Mink | adult | 2015.3 | Negative |
| 62 | Jilin | Jet Black Mink | adult | 2015.3 | Negative |
| 63 | Jilin | Jet Black Mink | adult | 2015.3 | Negative |
| 64 | Jilin | Jet Black Mink | adult | 2015.3 | Positive |
| 65 | Jilin | Jet Black Mink | adult | 2015.3 | Negative |
| 66 | Jilin | Jet Black Mink | adult | 2015.3 | Negative |
| 67 | Jilin | Jet Black Mink | adult | 2015.3 | Negative |
| 68 | Jilin | Jet Black Mink | adult | 2015.3 | Negative |
| 69 | Jilin | Jet Black Mink | adult | 2015.3 | Positive |
| 70 | Jilin | Jet Black Mink | adult | 2015.3 | Positive |
| 71 | Jilin | Jet Black Mink | adult | 2015.3 | Negative |
| 72 | Jilin | Jet Black Mink | adult | 2015.3 | Negative |
| 73 | Jilin | Jet Black Mink | adult | 2015.3 | Negative |
| 74 | Jilin | Jet Black Mink | adult | 2015.3 | Negative |
| 75 | Jilin | Jet Black Mink | adult | 2015.3 | Negative |
| 76 | Jilin | Jet Black Mink | adult | 2015.3 | Negative |
| 77 | Jilin | Jet Black Mink | adult | 2015.3 | Negative |
| 78 | Jilin | Jet Black Mink | adult | 2015.3 | Negative |
| 79 | Jilin | Jet Black Mink | adult | 2015.3 | Negative |
| 80 | Jilin | Jet Black Mink | adult | 2015.3 | Negative |
| 81 | Jilin | Jet Black Mink | adult | 2015.3 | Positive |
| 82 | Jilin | Jet Black Mink | adult | 2015.3 | Positive |
| 83 | Jilin | Jet Black Mink | adult | 2015.3 | Negative |
| 84 | Jilin | Jet Black Mink | adult | 2015.3 | Negative |
| 85 | Jilin | Jet Black Mink | adult | 2015.3 | Negative |
| 86 | Jilin | Jet Black Mink | adult | 2015.3 | Negative |
| 87 | Jilin | Jet Black Mink | adult | 2015.3 | Negative |
| 88 | Jilin | Jet Black Mink | adult | 2015.3 | Negative |
| 89 | Jilin | Jet Black Mink | adult | 2015.3 | Negative |
| 90 | Jilin | Jet Black Mink | adult | 2015.3 | Negative |
| 91 | Jilin | Jet Black Mink | adult | 2015.3 | Negative |
| 92 | Jilin | Jet Black Mink | adult | 2015.3 | Negative |
| 93 | Jilin | Jet Black Mink | adult | 2015.3 | Negative |
| 94 | Jilin | Jet Black Mink | adult | 2015.3 | Negative |
| 95 | Jilin | Jet Black Mink | adult | 2015.3 | Positive |
| 96 | Jilin | Jet Black Mink | adult | 2015.3 | Positive |
| 97 | Jilin | Jet Black Mink | adult | 2015.3 | Negative |
| 98 | Jilin | Jet Black Mink | adult | 2015.3 | Negative |
| 99 | Jilin | Jet Black Mink | adult | 2015.3 | Positive |
| 100 | Jilin | Jet Black Mink | adult | 2015.3 | Negative |
| 101 | Jilin | Jet Black Mink | adult | 2015.3 | Negative |
| 102 | Jilin | Jet Black Mink | adult | 2015.3 | Negative |
| 103 | Jilin | Jet Black Mink | adult | 2015.3 | Positive |
| 104 | Jilin | Jet Black Mink | adult | 2015.3 | Negative |
| 105 | Jilin | Jet Black Mink | adult | 2015.3 | Negative |
| 106 | Jilin | Jet Black Mink | adult | 2015.3 | Negative |
| 107 | Jilin | Jet Black Mink | adult | 2015.3 | Negative |
| 108 | Jilin | Jet Black Mink | adult | 2015.3 | Negative |
| 109 | Jilin | Jet Black Mink | adult | 2015.3 | Negative |
| 110 | Jilin | Jet Black Mink | adult | 2015.3 | Negative |
| 111 | Jilin | Jet Black Mink | adult | 2015.3 | Negative |
| 112 | Jilin | Jet Black Mink | adult | 2015.3 | Positive |
| 113 | Jilin | Jet Black Mink | adult | 2015.3 | Negative |
| 114 | Jilin | Jet Black Mink | adult | 2015.3 | Negative |
| 115 | Jilin | Jet Black Mink | adult | 2015.3 | Negative |
| 116 | Jilin | Jet Black Mink | adult | 2015.3 | Negative |
| 117 | Jilin | Jet Black Mink | adult | 2015.3 | Negative |
| 118 | Jilin | Jet Black Mink | adult | 2015.3 | Negative |
| 119 | Jilin | Jet Black Mink | adult | 2015.3 | Negative |
| 120 | Jilin | Jet Black Mink | adult | 2015.3 | Negative |
| 121 | Jilin | Jet Black Mink | adult | 2015.3 | Negative |
| 122 | Jilin | Jet Black Mink | adult | 2015.3 | Positive |
| 123 | Jilin | Jet Black Mink | adult | 2015.3 | Negative |
| 124 | Jilin | Jet Black Mink | adult | 2015.3 | Positive |
| 125 | Jilin | Jet Black Mink | adult | 2015.3 | Negative |
| 126 | Jilin | Jet Black Mink | adult | 2015.3 | Negative |
| 127 | Jilin | Jet Black Mink | adult | 2015.3 | Negative |
| 128 | Jilin | Jet Black Mink | adult | 2015.3 | Negative |
| 129 | Jilin | Jet Black Mink | adult | 2015.3 | Negative |
| 130 | Jilin | Jet Black Mink | adult | 2015.3 | Negative |
| 131 | Jilin | Jet Black Mink | adult | 2015.3 | Negative |
| 132 | Jilin | Jet Black Mink | adult | 2015.3 | Negative |
| 133 | Jilin | Jet Black Mink | adult | 2015.3 | Negative |
| 134 | Jilin | Jet Black Mink | adult | 2015.3 | Negative |
| 135 | Jilin | Jet Black Mink | adult | 2015.3 | Negative |
| 136 | Jilin | Jet Black Mink | adult | 2015.3 | Negative |
| 137 | Jilin | Jet Black Mink | adult | 2015.3 | Negative |
| 138 | Jilin | Jet Black Mink | adult | 2015.3 | Negative |
| 139 | Jilin | Jet Black Mink | adult | 2015.3 | Negative |
| 140 | Jilin | Jet Black Mink | adult | 2015.3 | Negative |
| 141 | Jilin | Jet Black Mink | adult | 2015.3 | Negative |
| 142 | Jilin | Jet Black Mink | adult | 2015.3 | Negative |
| 143 | Jilin | Jet Black Mink | adult | 2015.3 | Negative |
| 144 | Jilin | Jet Black Mink | adult | 2015.3 | Negative |
| 145 | Jilin | Jet Black Mink | adult | 2015.3 | Negative |
| 146 | Jilin | Jet Black Mink | adult | 2015.3 | Negative |
| 147 | Jilin | Jet Black Mink | adult | 2015.3 | Negative |
| 148 | Jilin | Jet Black Mink | adult | 2015.3 | Negative |
| 149 | Jilin | Jet Black Mink | adult | 2015.3 | Negative |
| 150 | Jilin | Jet Black Mink | adult | 2015.3 | Negative |
| 151 | Jilin | Jet Black Mink | adult | 2015.3 | Negative |
| 152 | Jilin | Jet Black Mink | adult | 2015.3 | Negative |
| 153 | Jilin | Jet Black Mink | adult | 2015.3 | Positive |
| 154 | Jilin | Jet Black Mink | adult | 2015.3 | Negative |
| 155 | Jilin | Jet Black Mink | adult | 2015.3 | Negative |
| 156 | Jilin | Jet Black Mink | adult | 2015.3 | Negative |
| 157 | Jilin | Jet Black Mink | adult | 2015.3 | Negative |
| 158 | Jilin | Jet Black Mink | adult | 2015.3 | Negative |
| 159 | Jilin | Jet Black Mink | adult | 2015.3 | Negative |
| 160 | Jilin | Jet Black Mink | adult | 2015.3 | Negative |
| 161 | Jilin | Jet Black Mink | adult | 2015.3 | Negative |
| 162 | Jilin | Jet Black Mink | adult | 2015.3 | Negative |
| 163 | Jilin | Jet Black Mink | adult | 2015.3 | Negative |
| 164 | Jilin | Jet Black Mink | adult | 2015.3 | Negative |
| 165 | Jilin | Jet Black Mink | adult | 2015.3 | Negative |
| 166 | Jilin | Jet Black Mink | adult | 2015.3 | Negative |
| 167 | Jilin | Jet Black Mink | adult | 2015.3 | Negative |
| 168 | Jilin | Jet Black Mink | adult | 2015.3 | Negative |
| 169 | Jilin | Jet Black Mink | adult | 2015.3 | Negative |
| 170 | Jilin | Jet Black Mink | adult | 2015.3 | Negative |
| 171 | Jilin | Jet Black Mink | adult | 2015.3 | Negative |
| 172 | Jilin | Jet Black Mink | adult | 2015.3 | Negative |
| 173 | Jilin | Jet Black Mink | adult | 2015.3 | Negative |
| 174 | Jilin | Jet Black Mink | adult | 2015.3 | Negative |
| 175 | Jilin | Jet Black Mink | adult | 2015.3 | Negative |
| 176 | Jilin | Jet Black Mink | adult | 2015.3 | Negative |
| 177 | Jilin | Jet Black Mink | adult | 2015.3 | Negative |
| 178 | Jilin | Jet Black Mink | adult | 2015.3 | Negative |
| 179 | Jilin | Jet Black Mink | adult | 2015.3 | Negative |
| 180 | Jilin | Jet Black Mink | adult | 2015.3 | Negative |
| 181 | Jilin | Jet Black Mink | adult | 2015.3 | Negative |
| 182 | Jilin | Jet Black Mink | adult | 2015.3 | Negative |
| 183 | Jilin | Jet Black Mink | adult | 2015.3 | Negative |
| 184 | Jilin | Jet Black Mink | adult | 2015.3 | Negative |
| 185 | Jilin | Jet Black Mink | adult | 2015.3 | Positive |
| 186 | Jilin | Jet Black Mink | adult | 2015.3 | Negative |
| 187 | Jilin | Jet Black Mink | adult | 2015.3 | Negative |
| 188 | Jilin | Jet Black Mink | adult | 2015.3 | Negative |
| 189 | Jilin | Jet Black Mink | adult | 2015.3 | Negative |
| 190 | Jilin | Jet Black Mink | adult | 2015.3 | Negative |
| 191 | Jilin | Jet Black Mink | adult | 2015.3 | Negative |
| 192 | Jilin | Jet Black Mink | adult | 2015.3 | Negative |
| 193 | Jilin | Jet Black Mink | adult | 2015.3 | Negative |
| 194 | Jilin | Jet Black Mink | adult | 2015.3 | Negative |
| 195 | Hebei | Jet Black Mink | adult | 2015.3 | Negative |
| 196 | Hebei | Jet Black Mink | adult | 2015.3 | Negative |
| 197 | Hebei | Jet Black Mink | adult | 2015.3 | Negative |
| 198 | Hebei | Jet Black Mink | adult | 2015.3 | Negative |
| 199 | Hebei | Jet Black Mink | adult | 2015.3 | Negative |
| 200 | Hebei | Jet Black Mink | adult | 2015.3 | Negative |
| 201 | Hebei | Jet Black Mink | adult | 2015.3 | Negative |
| 202 | Hebei | Jet Black Mink | adult | 2015.3 | Negative |
| 203 | Hebei | Jet Black Mink | adult | 2015.3 | Negative |
| 204 | Hebei | Jet Black Mink | adult | 2015.3 | Negative |
| 205 | Hebei | Jet Black Mink | adult | 2015.3 | Negative |
| 206 | Hebei | Jet Black Mink | adult | 2015.3 | Negative |
| 207 | Hebei | Jet Black Mink | adult | 2015.3 | Negative |
| 208 | Hebei | Jet Black Mink | adult | 2015.3 | Negative |
| 209 | Hebei | Jet Black Mink | adult | 2015.3 | Negative |
| 210 | Hebei | Jet Black Mink | adult | 2015.3 | Negative |
| 211 | Hebei | Jet Black Mink | adult | 2015.3 | Negative |
| 212 | Hebei | Jet Black Mink | adult | 2015.3 | Negative |
| 213 | Hebei | Jet Black Mink | adult | 2015.3 | Negative |
| 214 | Hebei | Jet Black Mink | adult | 2015.3 | Negative |
| 215 | Hebei | Jet Black Mink | adult | 2015.3 | Negative |
| 216 | Hebei | Jet Black Mink | adult | 2015.3 | Negative |
| 217 | Hebei | Jet Black Mink | adult | 2015.3 | Negative |
| 218 | Hebei | Jet Black Mink | adult | 2015.3 | Negative |
| 219 | Hebei | Jet Black Mink | adult | 2015.3 | Negative |
| 220 | Hebei | Jet Black Mink | adult | 2015.3 | Negative |
| 221 | Hebei | Jet Black Mink | adult | 2015.3 | Negative |
| 222 | Hebei | Jet Black Mink | adult | 2015.3 | Negative |
| 223 | Hebei | Jet Black Mink | adult | 2015.3 | Negative |
| 224 | Hebei | Jet Black Mink | adult | 2015.3 | Negative |
| 225 | Hebei | Jet Black Mink | adult | 2015.3 | Negative |
| 226 | Hebei | Jet Black Mink | adult | 2015.3 | Negative |
| 227 | Hebei | Jet Black Mink | adult | 2015.3 | Negative |
| 228 | Hebei | Jet Black Mink | adult | 2015.3 | Negative |
| 229 | Hebei | Jet Black Mink | adult | 2015.3 | Negative |
| 230 | Hebei | Jet Black Mink | adult | 2015.3 | Negative |
| 231 | Hebei | Jet Black Mink | adult | 2015.3 | Negative |
| 232 | Hebei | Jet Black Mink | adult | 2015.3 | Negative |
| 233 | Hebei | Jet Black Mink | adult | 2015.3 | Negative |
| 234 | Hebei | Jet Black Mink | adult | 2015.3 | Negative |
| 235 | Hebei | Jet Black Mink | adult | 2015.3 | Negative |
| 236 | Hebei | Jet Black Mink | adult | 2015.3 | Negative |
| 237 | Hebei | Jet Black Mink | adult | 2015.3 | Negative |
| 238 | Hebei | Jet Black Mink | adult | 2015.3 | Negative |
| 239 | Hebei | Jet Black Mink | adult | 2015.3 | Negative |
| 240 | Hebei | Jet Black Mink | adult | 2015.3 | Negative |
| 241 | Hebei | Jet Black Mink | adult | 2015.3 | Negative |
| 242 | Hebei | Jet Black Mink | adult | 2015.3 | Negative |
| 243 | Hebei | Jet Black Mink | adult | 2015.3 | Negative |
| 244 | Hebei | Jet Black Mink | adult | 2015.3 | Negative |
| 245 | Hebei | Jet Black Mink | adult | 2015.3 | Negative |
| 246 | Hebei | Jet Black Mink | adult | 2015.3 | Negative |
| 247 | Hebei | Jet Black Mink | adult | 2015.3 | Positive |
| 248 | Hebei | Jet Black Mink | adult | 2015.3 | Negative |
| 249 | Hebei | Jet Black Mink | adult | 2015.3 | Negative |
| 250 | Hebei | Jet Black Mink | adult | 2015.3 | Negative |
| 251 | Hebei | Jet Black Mink | adult | 2015.3 | Negative |
| 252 | Hebei | Jet Black Mink | adult | 2015.3 | Negative |
| 253 | Hebei | Jet Black Mink | adult | 2015.3 | Negative |
| 254 | Hebei | Jet Black Mink | adult | 2015.3 | Negative |
| 255 | Hebei | Jet Black Mink | adult | 2015.3 | Negative |
| 256 | Hebei | Jet Black Mink | adult | 2015.3 | Negative |
| 257 | Hebei | Jet Black Mink | adult | 2015.3 | Negative |
| 258 | Hebei | Jet Black Mink | adult | 2015.3 | Negative |
| 259 | Hebei | Jet Black Mink | adult | 2015.3 | Negative |
| 260 | Hebei | Jet Black Mink | adult | 2015.3 | Negative |
| 261 | Hebei | Jet Black Mink | adult | 2015.3 | Negative |
| 262 | Hebei | Jet Black Mink | adult | 2015.3 | Negative |
| 263 | Hebei | Jet Black Mink | adult | 2015.3 | Negative |
| 264 | Hebei | Jet Black Mink | adult | 2015.3 | Negative |
| 265 | Hebei | Jet Black Mink | adult | 2015.3 | Negative |
| 266 | Hebei | Jet Black Mink | adult | 2015.3 | Positive |
| 267 | Hebei | Jet Black Mink | adult | 2015.3 | Negative |
| 268 | Hebei | Jet Black Mink | adult | 2015.3 | Negative |
| 269 | Hebei | Jet Black Mink | adult | 2015.3 | Negative |
| 270 | Hebei | Jet Black Mink | adult | 2015.3 | Negative |
| 271 | Hebei | Jet Black Mink | adult | 2015.3 | Positive |
| 272 | Hebei | Jet Black Mink | adult | 2015.3 | Negative |
| 273 | Hebei | Jet Black Mink | adult | 2015.3 | Negative |
| 274 | Hebei | Jet Black Mink | adult | 2015.3 | Negative |
| 275 | Hebei | Jet Black Mink | adult | 2015.3 | Negative |
| 276 | Hebei | Jet Black Mink | adult | 2015.3 | Negative |
| 277 | Hebei | Jet Black Mink | adult | 2015.3 | Negative |
| 278 | Hebei | Jet Black Mink | adult | 2015.3 | Negative |
| 279 | Hebei | Jet Black Mink | adult | 2015.3 | Negative |
| 280 | Hebei | Jet Black Mink | adult | 2015.3 | Negative |
| 281 | Hebei | Jet Black Mink | adult | 2015.3 | Negative |
| 282 | Hebei | Jet Black Mink | adult | 2015.3 | Negative |
| 283 | Hebei | Jet Black Mink | adult | 2015.3 | Negative |
| 284 | Hebei | Jet Black Mink | adult | 2015.3 | Negative |
| 285 | Hebei | Jet Black Mink | adult | 2015.3 | Negative |
| 286 | Hebei | Jet Black Mink | adult | 2015.3 | Negative |
| 287 | Hebei | Jet Black Mink | adult | 2015.3 | Negative |
| 288 | Hebei | Jet Black Mink | adult | 2015.3 | Negative |
| 289 | Hebei | Jet Black Mink | adult | 2015.3 | Negative |
| 290 | Hebei | Jet Black Mink | adult | 2015.3 | Negative |
| 291 | Hebei | Jet Black Mink | adult | 2015.3 | Positive |
| 292 | Hebei | Jet Black Mink | adult | 2015.3 | Negative |
| 293 | Hebei | PalominoMink | adult | 2015.3 | Negative |
| 294 | Hebei | PalominoMink | adult | 2015.3 | Negative |
| 295 | Hebei | PalominoMink | adult | 2015.3 | Negative |
| 296 | Hebei | PalominoMink | adult | 2015.3 | Negative |
| 297 | Hebei | PalominoMink | adult | 2015.3 | Negative |
| 298 | Hebei | PalominoMink | adult | 2015.3 | Negative |
| 299 | Hebei | PalominoMink | adult | 2015.3 | Negative |
| 300 | Hebei | PalominoMink | adult | 2015.3 | Negative |
| 301 | Hebei | PalominoMink | adult | 2015.3 | Negative |
| 302 | Hebei | PalominoMink | adult | 2015.3 | Negative |
| 303 | Hebei | PalominoMink | adult | 2015.3 | Negative |
| 304 | Hebei | PalominoMink | adult | 2015.3 | Negative |
| 305 | Hebei | PalominoMink | adult | 2015.3 | Negative |
| 306 | Hebei | PalominoMink | adult | 2015.3 | Positive |
| 307 | Hebei | PalominoMink | adult | 2015.3 | Negative |
| 308 | Hebei | PalominoMink | adult | 2015.3 | Negative |
| 309 | Hebei | PalominoMink | adult | 2015.3 | Negative |
| 310 | Hebei | PalominoMink | adult | 2015.3 | Negative |
| 311 | Hebei | PalominoMink | adult | 2015.3 | Negative |
| 312 | Hebei | PalominoMink | adult | 2015.3 | Negative |
| 313 | Hebei | PalominoMink | adult | 2015.3 | Negative |
| 314 | Hebei | PalominoMink | adult | 2015.3 | Negative |
| 315 | Hebei | PalominoMink | adult | 2015.3 | Negative |
| 316 | Hebei | PalominoMink | adult | 2015.3 | Negative |
| 317 | Hebei | PalominoMink | adult | 2015.3 | Negative |
| 318 | Hebei | PalominoMink | adult | 2015.3 | Negative |
| 319 | Hebei | PalominoMink | adult | 2015.3 | Negative |
| 320 | Hebei | PalominoMink | adult | 2015.3 | Negative |
| 321 | Hebei | PalominoMink | adult | 2015.3 | Negative |
| 322 | Hebei | PalominoMink | adult | 2015.3 | Positive |
| 323 | Hebei | PalominoMink | adult | 2015.3 | Positive |
| 324 | Hebei | PalominoMink | adult | 2015.3 | Positive |
| 325 | Hebei | PalominoMink | adult | 2015.3 | Negative |
| 326 | Hebei | PalominoMink | adult | 2015.3 | Negative |
| 327 | Hebei | PalominoMink | adult | 2015.3 | Negative |
| 328 | Hebei | PalominoMink | adult | 2015.3 | Negative |
| 329 | Hebei | PalominoMink | adult | 2015.3 | Negative |
| 330 | Hebei | PalominoMink | adult | 2015.3 | Negative |
| 331 | Hebei | PalominoMink | adult | 2015.3 | Negative |
| 332 | Hebei | PalominoMink | adult | 2015.3 | Negative |
| 333 | Hebei | PalominoMink | adult | 2015.3 | Negative |
| 334 | Hebei | PalominoMink | adult | 2015.3 | Negative |
| 335 | Hebei | PalominoMink | adult | 2015.3 | Negative |
| 336 | Hebei | PalominoMink | adult | 2015.3 | Negative |
| 337 | Hebei | PalominoMink | adult | 2015.3 | Negative |
| 338 | Hebei | PalominoMink | adult | 2015.3 | Negative |
| 339 | Hebei | PalominoMink | adult | 2015.3 | Negative |
| 340 | Hebei | PalominoMink | adult | 2015.3 | Negative |
| 341 | Hebei | PalominoMink | adult | 2015.3 | Negative |
| 342 | Hebei | PalominoMink | adult | 2015.3 | Negative |
| 343 | Hebei | PalominoMink | adult | 2015.3 | Negative |
| 344 | Hebei | PalominoMink | adult | 2015.3 | Negative |
| 345 | Hebei | PalominoMink | adult | 2015.3 | Negative |
| 346 | Hebei | PalominoMink | adult | 2015.3 | Negative |
| 347 | Hebei | PalominoMink | adult | 2015.3 | Negative |
| 348 | Hebei | PalominoMink | adult | 2015.3 | Negative |
| 349 | Hebei | PalominoMink | adult | 2015.3 | Negative |
| 350 | Hebei | PalominoMink | adult | 2015.3 | Negative |
| 351 | Hebei | PalominoMink | adult | 2015.3 | Negative |
| 352 | Hebei | PalominoMink | adult | 2015.3 | Negative |
| 353 | Hebei | PalominoMink | adult | 2015.3 | Negative |
| 354 | Hebei | PalominoMink | adult | 2015.3 | Positive |
| 355 | Hebei | PalominoMink | adult | 2015.3 | Negative |
| 356 | Hebei | PalominoMink | adult | 2015.3 | Negative |
| 357 | Hebei | PalominoMink | adult | 2015.3 | Negative |
| 358 | Hebei | PalominoMink | adult | 2015.3 | Negative |
| 359 | Hebei | PalominoMink | adult | 2015.3 | Positive |
| 360 | Hebei | PalominoMink | adult | 2015.3 | Negative |
| 361 | Hebei | PalominoMink | adult | 2015.3 | Negative |
| 362 | Hebei | PalominoMink | adult | 2015.3 | Negative |
| 363 | Hebei | PalominoMink | adult | 2015.3 | Negative |
| 364 | Hebei | PalominoMink | adult | 2015.3 | Negative |
| 365 | Hebei | PalominoMink | adult | 2015.3 | Negative |
| 366 | Hebei | PalominoMink | adult | 2015.3 | Negative |
| 367 | Hebei | PalominoMink | adult | 2015.3 | Negative |
| 368 | Hebei | PalominoMink | adult | 2015.3 | Negative |
| 369 | Hebei | PalominoMink | adult | 2015.3 | Negative |
| 370 | Hebei | PalominoMink | adult | 2015.3 | Positive |
| 371 | Hebei | PalominoMink | adult | 2015.3 | Negative |
| 372 | Hebei | PalominoMink | adult | 2015.3 | Negative |
| 373 | Hebei | PalominoMink | adult | 2015.3 | Negative |
| 374 | Hebei | PalominoMink | adult | 2015.3 | Negative |
| 375 | Hebei | PalominoMink | adult | 2015.3 | Negative |
| 376 | Hebei | PalominoMink | adult | 2015.3 | Negative |
| 377 | Hebei | PalominoMink | adult | 2015.3 | Negative |
| 378 | Hebei | PalominoMink | adult | 2015.3 | Negative |
| 379 | Hebei | PalominoMink | adult | 2015.3 | Negative |
| 380 | Hebei | PalominoMink | adult | 2015.3 | Positive |
| 381 | Hebei | PalominoMink | adult | 2015.3 | Negative |
| 382 | Hebei | PalominoMink | adult | 2015.3 | Positive |
| 383 | Hebei | PalominoMink | adult | 2015.3 | Negative |
| 384 | Hebei | PalominoMink | adult | 2015.3 | Negative |
| 385 | Hebei | PalominoMink | adult | 2015.3 | Negative |
| 386 | Hebei | PalominoMink | adult | 2015.3 | Negative |
| 387 | Hebei | PalominoMink | adult | 2015.3 | Negative |
| 388 | Hebei | PalominoMink | adult | 2015.3 | Negative |
| 389 | Hebei | PalominoMink | adult | 2015.3 | Negative |
| 390 | Hebei | PalominoMink | adult | 2015.3 | Negative |
| 391 | Hebei | PalominoMink | adult | 2015.3 | Negative |
| 392 | Hebei | PalominoMink | adult | 2015.3 | Negative |
| 393 | Hebei | PalominoMink | adult | 2015.3 | Negative |
| 394 | Hebei | PalominoMink | adult | 2015.3 | Positive |
| 395 | Hebei | PalominoMink | adult | 2015.3 | Negative |
| 396 | Hebei | PalominoMink | adult | 2015.3 | Negative |
| 397 | Hebei | PalominoMink | adult | 2015.3 | Negative |
| 398 | Hebei | PalominoMink | adult | 2015.3 | Negative |
| 399 | Hebei | PalominoMink | adult | 2015.3 | Negative |
| 400 | Hebei | PalominoMink | adult | 2015.3 | Negative |
| 401 | Hebei | PalominoMink | adult | 2015.3 | Negative |
| 402 | Hebei | PalominoMink | adult | 2015.3 | Negative |
| 403 | Hebei | PalominoMink | adult | 2015.3 | Negative |
| 404 | Hebei | PalominoMink | adult | 2015.3 | Negative |
| 405 | Hebei | PalominoMink | adult | 2015.3 | Negative |
| 406 | Hebei | PalominoMink | adult | 2015.3 | Negative |
| 407 | Hebei | PalominoMink | adult | 2015.3 | Positive |
| 408 | Hebei | PalominoMink | adult | 2015.3 | Negative |
| 409 | Hebei | PalominoMink | adult | 2015.3 | Negative |
| 410 | Hebei | PalominoMink | adult | 2015.3 | Positive |
| 411 | Hebei | PalominoMink | adult | 2015.3 | Positive |
| 412 | Hebei | PalominoMink | adult | 2015.3 | Negative |
| 413 | Hebei | PalominoMink | adult | 2015.3 | Negative |
| 414 | Hebei | PalominoMink | adult | 2015.3 | Negative |
| 415 | Hebei | PalominoMink | adult | 2015.3 | Negative |
| 416 | Hebei | PalominoMink | adult | 2015.3 | Negative |
| 417 | Hebei | PalominoMink | adult | 2015.3 | Positive |
| 418 | Hebei | PalominoMink | adult | 2015.3 | Positive |
