# Supplementary material for: Molecular Detection and Genetic Characterization of Toxoplasma gondii in Farmed Minks (Neovison vison) in Northern China by PCR-RFLP
Source: PLoS One. 2016 Nov 2;11(11):e0165308. doi: 10.1371/journal.pone.0165308 (PMC5091863; doi:10.1371/journal.pone.0165308)
Supplement: S2 Table — (DOCX) [file pone.0165308.s002.docx]

**S2 Table.** Nested PCR primers used for nested PCR-RFLP of *Toxoplasma gondii* in minks.

|  | Sequence (5'→3')^a^ | |
| --- | --- | --- |
| locus | External primers | Internal primers |
| SAG1 | F: GTTCTAACCACGCACCCTGAG | F: CAATGTGCACCTGTAGGAAGC |
|  | R: AAGAGTGGGAGGCTCTGTGA | R: GTGGTTCTCCGTCGGTGTGAG |
|  | | |
| 5'-SAG2 | F: GCTACCTCGAACAGGAACAC | F: GAAATGTTTCAGGTTGCTGC |
|  | R: GCATCAACAGTCTTCGTTGC | R: GCAAGAGCGAACTTGAACAC |
|  | | |
| 3'-SAG2 | F: TCTGTTCTCCGAAGTGACTCC | F: ATTCTCATGCCTCCGCTTC |
|  | R: TCAAAGCGTGCATTATCGC | R: AACGTTTCACGAAGGCACAC |
|  | | |
| alter.SAG2 | F: GGAACGCGAACAATGAGTTT | F: ACCCATCTGCGAAGAAAACG |
|  | R: GCACTGTTGTCCAGGGTTTT | R: ATTTCGACCAGCGGGAGCAC |
|  | | |
| SAG3 | F: CAACTCTCACCATTCCACCC | F: TCTTGTCGGGTGTTCACTCA |
|  | R: GCGCGTTGTTAGACAAGACA | R: CACAAGGAGACCGAGAAGGA |
|  | | |
| BTUB | F: TCCAAAATGAGAGAAATCGT | F: GAGGTCATCTCGGACGAACA |
|  | R: AAATTGAAATGACGGAAGAA | R: TTGTAGGAACACCCGGACGC |
|  | | |
| GRA6 | F: ATTTGTGTTTCCGAGCAGGT | F: TTTCCGAGCAGGTGACCT |
|  | R: GCACCTTCGCTTGTGGTT | R: TCGCCGAAGAGTTGACATAG |
|  | | |
| c22-8 | F: TGATGCATCCATGCGTTTAT | F: TCTCTCTACGTGGACGCC |
|  | R: CCTCCACTTCTTCGGTCTCA | R: AGGTGCTTGGATATTCGC |
|  | | |
| c29-2 | F: ACCCACTGAGCGAAAAGAAA | F: AGTTCTGCAGAGTGTCGC |
|  | R: AGGGTCTCTTGCGCATACAT | R: TGTCTAGGAAAGAGGCGC |
|  | | |
| L358 | F: TCTCTCGACTTCGCCTCTTC | F: AGGAGGCGTAGCGCAAGT |
|  | R: GCAATTTCCTCGAAGACAGG | R: CCCTCTGGCTGCAGTGCT |
|  | | |
| PK1 | F: GAAAGCTGTCCACCCTGAAA | F: CGCAAAGGGAGACAATCAGT |
|  | R: AGAAAGCTCCGTGCAGTGAT | R: TCATCGCTGAATCTCATTGC |
|  | | |
| Apico | F: TGGTTTTAACCCTAGATTGTGG | F: GCAAATTCTTGAATTCTCAGTT |
|  | R: AAACGGAATTAATGAGATTTGAA | R: GGGATTCGAACCCTTGATA |

a F, forward primer; R, reverse primer.
